# Differential response in levels of high-density lipoprotein cholesterol to one-year metformin treatment in prediabetic patients by race/ethnicity

**DOI:** 10.1186/s12933-015-0240-1

**Published:** 2015-06-12

**Authors:** Chao Zhang, Feng Gao, Hao Luo, Chun-Ting Zhang, Ren Zhang

**Affiliations:** Division of Geriatric and Palliative Medicine, School of Medicine, University of Michigan, Ann Arbor, MI USA; Department of Physics, Tianjin University, Tianjin, China; Center for Molecular Medicine and Genetics, School of Medicine, Wayne State University, Detroit, MI 48201 USA; Cardiovascular Research Institute, School of Medicine, Wayne State University, Detroit, MI USA

**Keywords:** Cholesterol, Diabetes, HDL, Metformin

## Abstract

**Background:**

As a first-line diabetes drug that is widely prescribed around the world, metformin has been demonstrated to be effective in reducing microvascular risk, in addition to lowering glucose levels. Specifically, metformin use has been shown to be associated with improved lipid profiles, such as increased levels of high-density lipoprotein cholesterol (HDL-C). However, no study has been performed to examine the differential response in HDL-C levels to metformin treatment by race/ethnicity.

**Methods:**

Here, based on a re-analysis of the data from the Diabetes Prevention Program, which involved pre-diabetic participants receiving 850 mg of metformin twice daily, we compared the lipid profile changes following the metformin use. The participants were composed of 602 Whites, 221 African Americans (AAs) and 162 Hispanics.

**Results:**

We found that the one-year metformin treatment resulted in a significant increase in HDL-C levels in Whites (*p* = 0.002) and AAs (*p* = 0.016), but not in Hispanics. Consistently, both Whites (*p* = 0.018) and AAs (*p* = 0.020) had more pronounced changes in HDL-C levels than Hispanics following metformin treatment.

**Conclusion:**

This result suggests a notion that Whites and AAs are more responsive than Hispanics to one-year metformin use in HDL-C level changes, and that racial and ethnic identity is a factor to consider when interpreting the effects of metformin treatment on lipid profiles.

## Background

As a first-line drug for treating type 2 diabetes, metformin has been demonstrated to be effective in glycemic control [1], and is thus widely prescribed around the world. In the United States, metformin is the most commonly prescribed diabetes medication in 2010, with more than 48 million prescriptions being filled [2]. Metformin has been shown to reduce microvascular risk [1, 3-5], in addition to producing beneficial effects in other diseases involving insulin resistance, such as polycystic ovary syndrome [6] and non-alcoholic fatty liver disease [7].

Diabetes is commonly associated with abnormality in lipid profiles, characterized by increased triglycerides, reduced HDL-C, and postprandial lipemia, known as diabetic dyslipidemia [8-11]. Cardiovascular morbidity and mortality are higher in patients with type 2 diabetes than in the general population [8, 12, 13], partly due to accelerated development of atherosclerosis [14, 15]. In fact, the first cause of death in diabetic patients is heart disease or stroke. Indeed, diabetes treatment can reduce the chances of heart disease and improve cardiovascular outcome [16, 17]. Specifically, metformin treatments have been shown to be associated with improved lipid profiles, such as reduced triglycerides and increased HDL-C [18-25].

Because of the widespread use of metformin in the world and the high prevalence of diabetes among different racial and ethnic groups, it is important to examine the racial difference in response to metformin treatments. Indeed, it was recently shown that African Americans (AAs) have a better glycemic response to metformin compared to European Americans [26]. Nevertheless, no study has been performed to examine the differential response in HDL-C levels following metformin treatment by race/ethnicity. Here, based on the re-analysis of the data from the Diabetes Prevention Program (DPP) [27, 28], we compared the lipid profile changes after one-year treatment of metformin. We found that in the metformin group, but not in the placebo group, one-year metformin increased HDL-C levels in Whites and AAs, but not in Hispanics.

## Materials and methods

The DPP data Archive version 2.1 was obtained from the National Institute of Diabetes and Digestive and Kidney (NIDKK) data repository. The DPP was a multi-center clinical trial examining effects of intensive lifestyle changes or metformin versus placebo on the rate of developing diabetes. Qualified subjects all had prediabetes, due to elevated fasting glucose, impaired glucose tolerance, and obesity. Informed consent was obtained from all subjects. The metformin group received 850 mg of metformin twice a day. Of note, the placebo groups also received information about diet and exercise but no intensive motivational counseling. Forty five percent of the participants were recruited from minority groups, such as African American, Alaska Native, American Indian, Asian American, and Hispanics, who have an increased risk of developing diabetes [29]. Lipid profile measurements and lipoprotein subclass particle concentration determination were performed as previously described [30].

Student *t* tests and ANOVA were performed to compare differences in lipid levels among Whites, AAs and Hispanics, and paired *t* tests were performed to compare differences in lipid levels between baseline and year-one values. A generalized estimating equation was implemented to examine the rate of change in HDL-C levels in response to metformin treatment, and it was adjusted for confounding factors including age, gender, body mass index, triglycerie, LDL-C, cholesterol and fasting glucose levels. Average mean change from baseline to year 1 among Whites, AAs, and Hispanic were computed using One-way ANOVA, and Dunnett method was performed for multiple comparison tests. The Software SAS 9.3 (SAS Institute, Cary, NC) was used to perform statistical tests, and *P* < 0.05 was considered significant. The study protocol was approved by the Institutional Review Boards of Wayne State University School of Medicine and of the Detroit Medical Center, and all studies were carried out in accordance with the approved guidelines.

## Results

### HDL-C levels are increased in the metformin group, but not in the placebo group

The current study included 985 participants, made up of 650 females and 335 males. The distributions of age and BMI are listed in Table 1. All DPP participants were pre-diabetic. Consistently, at baseline, the fasting plasma glucose and insulin levels were 107.25 ± 7.84 (mg/dL) and 26.99 ± 14.80 (μIU/mL), respectively, and the HbA1C level was 5.91 ± 0.51 (%).

**Table 1:** Clinical characteristics of the DPP participants treated with metformin by racial/ethnic group

	Whites	African Americans	Hispanics	All
N	602	221	162	985
Male	225	58	52	335
Female	377	163	110	650
Age (years)				
< 40	65	27	36	128
40 – 49	203	79	56	338
50 – 59	197	70	50	317
> = 60	137	45	20	202
BMI (kg/m^2^)				
< 30	181	87	45	313
30 to < 36	227	71	63	361
> = 36	194	63	54	311
Fasting plasma glucose (mg/dL)	107.05 ± 7.64	108.35 ± 8.00	106.49 ± 8.21	107.25 ± 7.84
Fasting plasma insulin (μIU/mL)	26.03 ± 14.92	28.93 ± 15.76	27.93 ± 12.62	26.99 ± 14.80
HbA1c (%)	5.80 ± 0.43	6.20 ± 0.58	5.89 ± 0.51	5.91 ± 0.51

Data for levels of plasma glucose, insulin and HbA1c are presented as means ± SD

*BMI* body mass index

In the metformin group, the baseline and year-one HDL-C levels (mg/dL) were 46.17 ± 11.58 and 46.88 ± 12.07, respectively. Compared to the baseline level, the year-one level was significantly increased, with the average increase being 0.67 ± 6.49 (paired *t* test, *p =* 0.002). In the placebo group, the HDL-C levels (mg/dL) at baseline and year one were: 44.79 ± 11.51 and 44.78 ± 11.41, respectively; and the average change (−0.09 ± 6.05) between the baseline and year one was not significant (*p =* 0.639) (Table 2). Therefore, HDL-C levels were increased in the metformin group, but not in the placebo group.

**Table 2:** Lipid profiles in DPP participants by treatment group at baseline and one-year follow-up

Group	N (female)	Variable	Baseline	Year 1	Delta	*P* value^a^
Metformin	985 (650)	TRIG	157.02 ± 86.87	152.59 ± 90.99	−5.01 ± 67.09	0.023
		CHOL	202.96 ± 35.25	198.50 ± 34.88	−4.62 ± 25.18	<0.001
		HDL-C	46.17 ± 11.58	46.88 ± 12.07	0.67 ± 6.49	0.002
		VLDL	31.26 ± 16.93	30.40 ± 17.85	−0.98 ± 13.45	0.027
		CLDL	125.05 ± 32.06	120.79 ± 31.14	−4.27 ± 23.08	<0.001
		LDLB	69.58 ± 17.67	67.97 ± 17.49	−1.59 ± 13.86	<0.001
		LDLC	107.67 ± 27.08	103.92 ± 26.95	−3.65 ± 20.98	<0.001
		LDLZ	0.27 ± 0.03	0.27 ± 0.03	0.00 ± 0.02	0.761
Placebo	973 (675)	TRIG	167.96 ± 93.87	159.07 ± 95.36	−9.25 ± 73.84	<0.001
		CHOL	203.62 ± 36.35	199.67 ± 35.07	−4.04 ± 26.64	<0.001
		HDL-C	44.79 ± 11.51	44.78 ± 11.41	−0.09 ± 6.05	0.639
		VLDL	33.31 ± 17.73	31.66 ± 18.47	−1.70 ± 13.97	<0.001
		CLDL	125.04 ± 33.47	122.76 ± 31.21	−2.22 ± 24.67	0.006
		LDLB	69.56 ± 18.38	68.44 ± 17.36	−1.15 ± 13.51	0.01
		LDLC	106.48 ± 28.38	103.78 ± 27.04	−2.65 ± 21.51	<0.001
		LDLZ	0.26 ± 0.03	0.26 ± 0.03	0.00 ± 0.03	0.830

*CHOL,* cholesterol; *HDL-C,* high density lipoprotein cholesterol; *LDL,* low density lipoprotein cholesterol; *LDLB, LDL-B* sub-fraction; *LDLC, LDL-C* sub-fraction; *LDLZ, LDL* particle size; *TRIG*, triglycerides; *VLDL*, very low density lipoprotein cholesterol. Data are presented as mean ± SD

^a^ Paired Student *t* test for difference between baseline and year-one values. The original abbreviations in the DPP dataset were adopted

We next examined other lipid parameters following metformin treatment (Table 2). In the metformin group, compared to the baseline values, levels of triglycerides, total cholesterol, VLDL and LDL-C were all significantly reduced at year one, with changes (mg/dL) being −5.01 ± 67.09 (*p =* 0.023), −4.62 ± 25.18 (*p <* 0.001), −0.98 ± 13.45 (*p =* 0.027) and −4.27 ± 23.08 (*p <* 0.001), respectively. Interestingly, at year one in the placebo group, the levels of triglycerides, total cholesterol, VLDL and LDL-C were significantly reduced as well, with changes being −9.25 ± 73.84 (*p <*0.001), −4.04 ± 26.64 (*p <* 0.001), −1.70 ± 13.97 (*p <* 0.001) and −2.22 ± 24.67 (*p =* 0.006), respectively (Table 2).

We also examined the LDL-subfraction changes in both metformin and placebo groups. Compared to the baseline values, in the metformin group, both LDL B subfraction (−1.59 ± 13.86, *p <* 0.001) and C subfraction (−3.65 ± 20.98, *p <* 0.001) were decreased at year 1. Likewise, in the placebo group, both LDL B and C subfractions were decreased as well, with changes being −1.15 ± 13.51 (*p <* 0.001) and −2.65 ± 21.51 (*p <* 0.001), respectively. In contrast, the LDL particle size was not significantly changed in both metformin and placebo groups. Therefore, among different parameters in the lipid profile, only changes in HDL-C levels were distinct between the metformin group and the placebo group, suggesting that one-year metformin treatment to be the factor that increased HDL cholesterol levels.

### One-year metformin increases HDL cholesterol in Whites and African Americans, but not in Hispanics

We next examined the HDL-C levels following metformin treatment among different race/ethnicity. In the metformin group, there were 602 Whites, 221 AAs, and 162 Hispanics, among which 377, 163, and 110 were women, respectively (Table 1). Distribution of ages and BMIs are listed in Table 1 for different race/ethnicity.

**Fig. 1:**
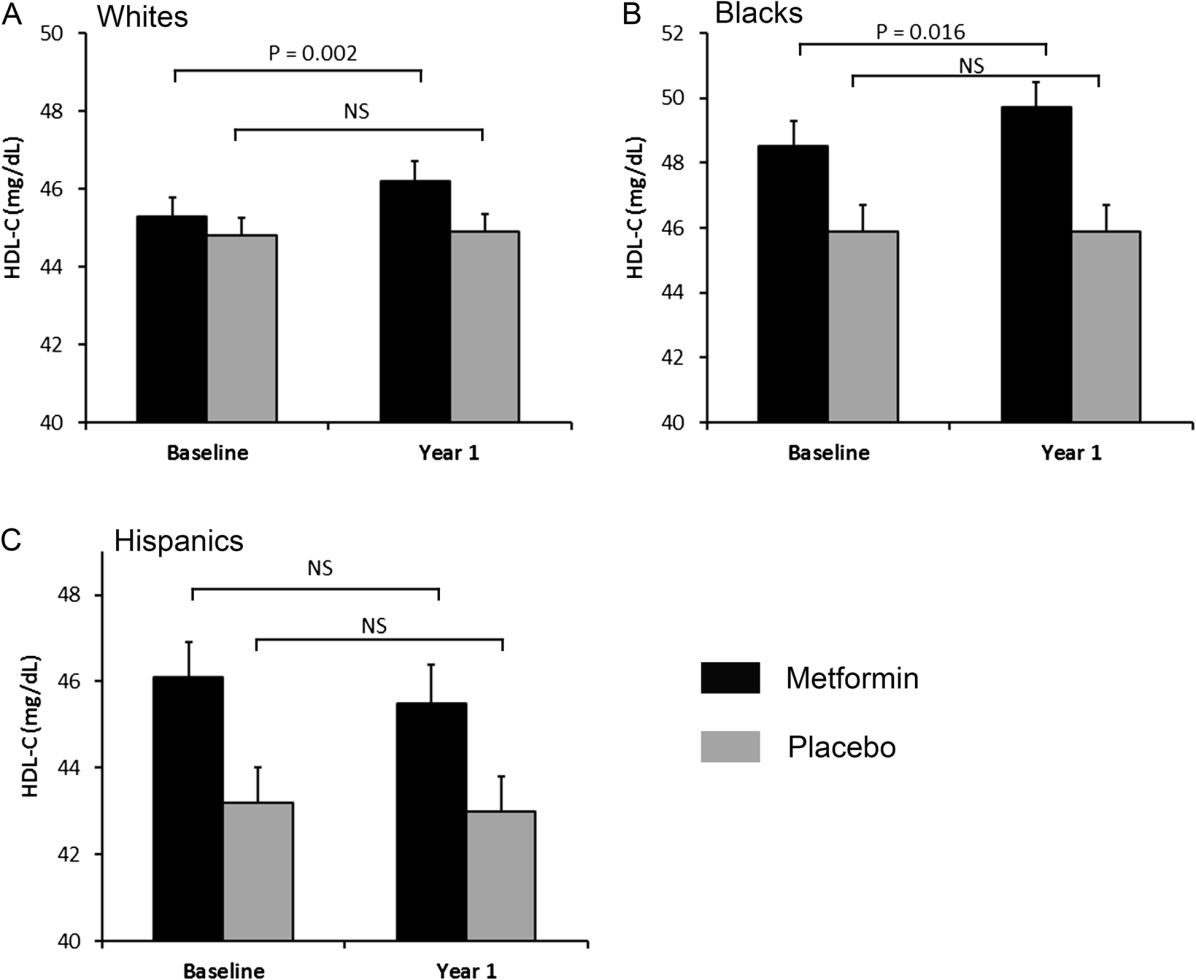
One-year metformin treatment increases HDL cholesterol in Whites and African Americans, but not in Hispanics. Levels of HDL-C at baseline and one-year checkup in **a** Whites, **b** African Americans and **c** Hispanics. Paired Student *t* tests were performed to compare HDL-C levels in the baseline and year-one checkup. HDL-C, high-density lipoprotein cholesterol; NS, non-significant. Data are represented as mean ± SEM

The HDL-C levels in the placebo group at baseline and year one in Whites were (mg/dL, mean ± SEM) 44.8 ± 0.47 and 44.9 ± 0.47, respectively; in AAs were 45.9 ± 0.81 and 45.9 ± 0.87, respectively; in Hispanics were 43.2 ± 0.85 and 43.0 ± 0.84, respectively (Fig. 1). The HDL-C levels in the metformin group at baseline and year one in Whites were 45.3 ± 0.48 and 46.2 ± 0.51, respectively, in AAs were 48.5 ± 0.80 and 49.7 ± 0.87, respectively, and in Hispanics were 46.1 ± 0.80 and 45.5 ± 0.89, respectively (Fig. 1).

We performed paired Student *t* tests to compare HDL-C levels in the baseline and year-one checkup. In the metformin group, one-year metformin treatment resulted in significant increases in HDL-C levels in Whites (*p =* 0.002) and AAs (*p =* 0.016), but not in Hispanics (*p =* 0.329) (Fig. 1). In contrast, in the placebo group, there were no significant changes in levels of HDL-C for Whites (*p =* 0.564), AAs (*p =* 0.902), as well as for Hispanics (*p =* 0.845). These results suggest that one-year metformin treatment increased HDL-C levels in Whites, AAs, but not in Hispanics.

### Differences in change of HDL-C levels among participants with different race/ethnicity

**Fig. 2:**
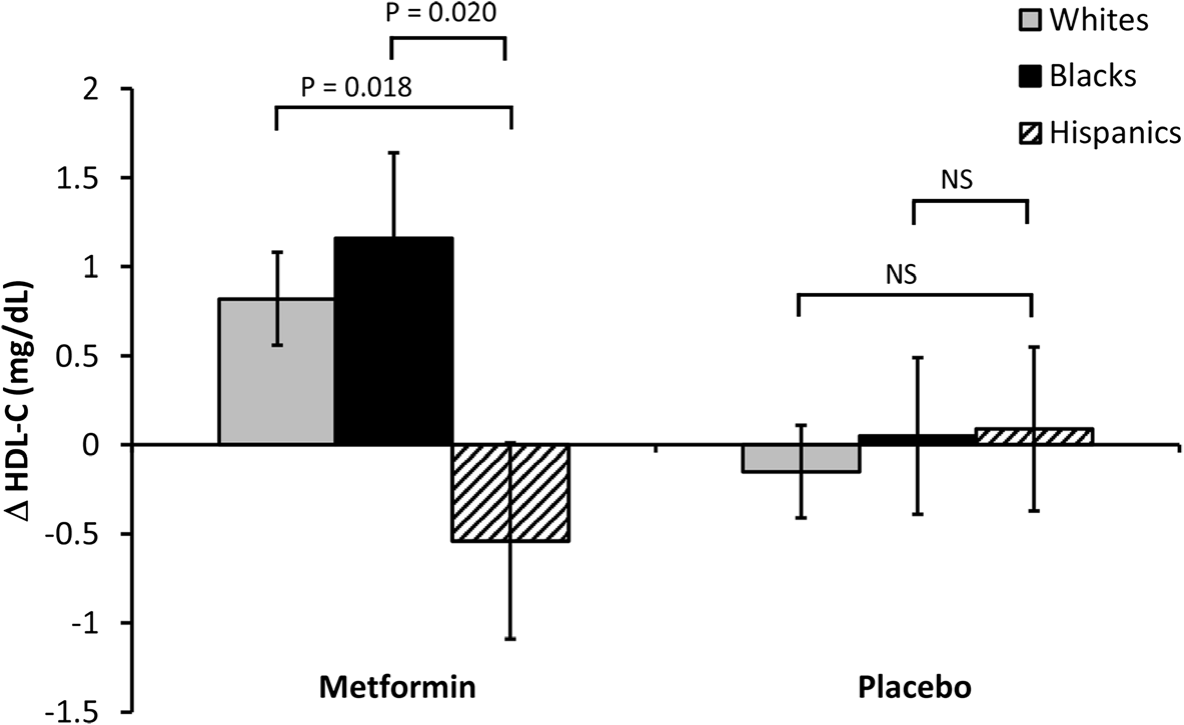
Changes in HDL-C in response to one-year metformin treatment among races. Changes = Value_one-year_ – value_baseline_. *P* values were obtained by performing Student *t* tests. HDL-C, high-density lipoprotein cholesterol. Data are represented as mean ± SEM

We next examined the changes in HDL-C levels before and after one-year metformin treatment in both placebo and metformin groups among race/ethnicity. The average changes between baseline and year one for Whites, AAs, and Hispanics were 0.82 ± 0.26, 1.16 ± 0.48, and −0.54 ± 0.55, respectively. In the placebo group, the average changes between baseline and year one for Whites, AAs, and Hispanics were 0.15 ± 0.26, 0.05 ± 0.44, and 0.09 ± 0.46, respectively. When comparing HDL-C level changes, in the metformin group, Whites had a more dramatic change in HDL-C levels than Hispanics (*p =* 0.018), but in the placebo group, there was no significant difference between Whites and Hispanics (*p =* 0.916) (Fig. 2). Likewise, in the metformin group, AAs had a more dramatic change in HDL-C levels than Hispanics (*p =* 0.020), but in the placebo group, there was no significant difference between Whites and Hispanics (*p =* 0.823) (Fig. 2). Therefore, both Whites and AAs had more dramatic HDL-C level changes than Hispanics by one-year metformin treatment, but in the placebo group, neither Whites nor AAs had more HDL-C level changes than Hispanics.

To confirm these results, we also performed One-way ANOVA to compare the changes in HDL-C in both metformin and placebo groups among race/ethnicity. For metformin treatment, the F value and the *p* value were 3.48 and 0.031, respectively. We also took Hispanics as reference and performed multiple comparison tests, the Dunnett method. The difference of change between Whites and Hispanics was 1.70 (95 % CI, 0.20–3.20, *p <* 0.05), and the difference of change between AAs and Hispanics was 1.36 (95 % CI, 0.08–2.64, *p* < 0.05). For the placebo group, the F value and the *p* value were 0.08 and 0.920, respectively, by performing One-way ANOVA. Therefore, both Whites and AAs had more dramatic HDL-C level changes than Hispanics by one-year metformin treatment, but in the placebo group, neither Whites nor AAs had more HDL-C level changes than Hispanics.

## Discussion

It was well established that HDL-C levels are associated with other metabolism parameters, such as triglyceride and LDL-C levels [31-34]. In addition, patients with type 2 diabetes are commonly associated with diabetic dyslipidemia, characterized by high levels of triglyceride and LDL-C, but low levels of HDL-C [10, 35-37]. It was found that in type 2 diabetes, HDL carries higher level of sphingosine-1-phosphate, which has the potential to contribute to protective effects on endothelial cells by inducing the expression of cyclooxygenase 2 [38]. Another potential mechanism is through vitamin B12 deficiency, which is common in type 2 diabetes, and is associated with lipid profiles [39]. The association between insulin sensitivity and lipid profiles is even present in heathy subjects. For instance, it was found that in healthy hyperalphalipoproteinemia subjects, several parameters that control the metabolism of plasma cholesterol and lipoproteins are related to a higher degree of insulin sensitivity [36].

Because of the association between HDL-C levels and other metabolic parameters, it is important to exclude the effects of potential confounding factors. In statistics, a generalized estimating equation (GEE) is used to estimate the parameters of a generalized linear model with a possible unknown correlation between outcomes for longitudinal data, and regression models are useful to adjust for confounders. That is, models with covariates can detect the effects of each covariate while the effects of other covariates being partialed out [40]. We therefore performed the GEE model to confirm whether the change rate of HDL-C level over time for Whites and AAs was greater than that of Hispanics in patients with metformin treatment. As listed in Table 3, based on the unadjusted model, the slopes (Year*Race) for Whites and AAs were 1.37 and 1.71, respectively. It means that the change rates of HDL-C from the baseline to year one were 1.37 mg/dL (*p* = 0.024) and 1.71 mg/dL (*p* = 0.019) greater than that of Hispanics. Consistently, after adjusting for covariates including age, gender, BMI, levels of triglyceride, cholesterol, LDL-C and fasting glucose, the rates in HDL-C changes for Whites (1.29, *p* = 0.022) and AAs (1.38, *p* = 0.039) were still significantly greater than that of Hispanics. Therefore, the observation that Whites and AAs were more sensitive towards metformin treatment in HDL-C levels than Hispanics is unlikely due to the potential confounding factors.

**Table 3:** Results of unadjusted and adjusted GEE modes for subjects with metformin treatment

Parameter	Unadjusted model			Adjusted model^a^	
Time*Race		Estimate (95 % CI)	*P* value	Estimate (95 % CI)	*P* value
	White	1.37 (0.18–2.56)	0.024	1.29 (0.19–2.38)	0.022
	AA	1.71 (0.29–3.13)	0.019	1.38 (0.07–2.69)	0.039
	Hispanic	-	-	-	-

CI, confidence interval; GEE, generalized estimating equation

^a^ Adjusted for age, gender, body mass index, triglyceride, cholesterol, LDL-C and fasting glucose levels

One limitation of the current study is that the DPP was not designed to examine racial differences under interventions of metformin and life style change, and therefore it lacked the statistical power for such analysis. To obtain significant results, we thus pooled the data from both male and female participants, and therefore it is unknown whether the observed differences were gender specific. Another limitation is that the current study focuses on lipid profiles by one-year metformin treatment, but HDL-C differences among races in response to metformin appeared to diminish over time. Furthermore, all DPP participants were prediabetic, and therefore whether the observed racial differences can be applied to patients with type 2 diabetes or other metabolic syndrome is uncertain. In addition, the numbers of studied subjects with different races were not well balanced, i.e., there were 602 Whites, 221 AAs and 162 Hispanics. Although the number of 162 in Hispanics can be considered large enough to reach statistical significance in change of HDL-C levels, if any, it will be necessary to confirm the observation in future studies using cohorts composed of relatively large number of Hispanics. In addition, because of the limited number of Asians in the DPP, Asians were not included in the current analysis.

To the best of our knowledge, this is the first report on the differential response in lipid profile changes to metformin treatment by race/ethnicity. Therefore, future studies need to confirm the current observation based on independent cohorts. Specifically, it would be important for future studies to include more time points, different metformin doses, different races, and different disease conditions, such as type 2 diabetes and polycystic ovary syndrome. It would be also interesting to examine the race-ethnicity difference in response to other diabetic treatments, which may be associated with distinct lipid profile changes [41]. Another important direction is to identify the molecular mechanisms responsible for the racial difference in response to metformin, and the identification of these mechanisms has the potential for new development to increase the drug potency.

## Conclusion

In conclusion, we found that in the prediabetic population, a one-year metformin treatment raised HDL-C in Whites and AAs, but not in Hispanics in the DPP. This result suggests differences of HDL-C levels in response to metformin by race/ethnicity, and that racial and ethnic identity is a factor to consider when interpreting the effects of metformin treatment.

## Abbreviations

AA: African American
CHOL: Cholesterol
DPP: Diabetes Prevention Program
GEE: Generalized estimating equation
LDL: Low density lipoprotein cholesterol
HDL-C: High density lipoprotein cholesterol
LDLB: LDL-B sub-fraction
LDLC: LDL-C sub-fraction
LDLZ: LDL particle size
TRIG: Triglycerides
VLDL: Very low density lipoprotein cholesterol
